# Gene–Nutrition Interaction in Gastric Cancer: miRNA-Related Polymorphisms in *CTSF* and *NT5E*, Dietary Exposures, and Site-Specific Risk

**DOI:** 10.3390/nu18213574

**Published:** 2026-10-29

**Authors:** Dan Wang, Shilin Chen, Yalan Li, Ruoyu Li, Peifang Liu, Yu Jiang, Jianjun Xiang, Wei Yan, Chuancheng Wu

**Affiliations:** 1 Department of Preventive Medicine, School of Public Health, Fujian Medical University, Fuzhou 350122, China; 2 Key Laboratory of Environment and Health, Fujian Province University, Fuzhou 350122, China; 3 The First Hospital of Putian City, Putian 351100, China

**Keywords:** gastric cancer, gastrointestinal diseases, miRNA-related polymorphism, gene–diet interaction, *CTSF*, *NT5E*

## Abstract

Background: Gastric cancer (GC) is a leading cause of cancer death in China, particularly in rural high-incidence areas. miRNA-related SNPs may interact with diet to influence GC risk. We therefore examined miRNA-related SNPs in *COPS*2, *CTSF*, and *NT5E*, together with their cognate miRNAs, and assessed their interactions with dietary factors in overall, cardia, and non-cardia GC. Methods: A 1:1 matched case-control study included 550 GC cases and 550 controls from a high-incidence county in Fujian, China. Eight miRNA-related SNPs were genotyped, and seven conforming to Hardy–Weinberg equilibrium were included in the final analysis. Conditional logistic regression and generalized multifactor dimensionality reduction (GMDR) were used to assess associations and interactions. Results: *CTSF* rs4576 was associated with increased overall and non-cardia GC risk. *NT5E* rs6913634 was associated with increased overall and cardia G*C* risk, whereas miR-30a rs2222722 was associated with increased risk of cardia GC only. GMDR identified hard-food preference, pickled vegetable intake, and rs4576 as the best interaction model for overall GC. A positive additive interaction was observed between rs4576 and hard-food preference (RERI = 1.65, 95% CI: 0.049–3.26). G-allele carriers with hard-food preference or pickled vegetable intake had markedly elevated joint risks. Conclusions: miRNA-related polymorphisms in *CTSF* and *NT5E* and in miR-30a showed subsite-related association patterns in this high-risk Chinese population; however, without formal between-subsite heterogeneity tests, these should be interpreted as observed patterns rather than established subtype-specific differences. Additionally, the preliminary positive additive interaction between rs4576 and hard-food preference points to a potential gene–diet interaction that warrants validation in independent populations before it can inform dietary prevention.

## 1. Introduction

Gastric cancer (GC) is the fifth most common cancer and the fifth leading cause of cancer death worldwide [1]. In 2022, there were an estimated 969,000 new GC cases and 660,000 GC-related deaths globally, of which China accounted for 37% of the new diagnoses [1]. In China, GC continues to pose a substantial public health challenge: the age-standardized incidence rate (ASIR) and mortality rate (ASMR) in 2022 were 13.7 and 9.4 per 100,000, respectively, with corresponding crude rates of 25.4 and 18.4 per 100,000 [2]. Age-standardized incidence and mortality have declined gradually since 2018, reflecting the cumulative effects of *H. pylori* eradication, improved food preservation, and strengthened primary prevention [2,3]; nevertheless, because of the large and rapidly ageing population [4], the absolute numbers of cases and deaths continue to rise. Moreover, the majority of Chinese patients are diagnosed at advanced stages, when five-year survival remains poor and treatment costs are among the highest for digestive malignancies [5].

Gastric carcinogenesis is a multistage process involving environmental, infectious, lifestyle, and inherited factors. Chronic infection with *H. pylori*, an IARC Group 1 carcinogen, is the dominant infectious risk factor for non-cardia GC [6]. Tobacco smoking and excessive alcohol consumption promote carcinogenesis through oxidative stress and repeated mucosal injury [7]. Most relevant to the present study, diets high in salt and salt-preserved or pickled vegetables also drive this process via intragastric nitrosation; this mucosal injury may be further aggravated by the hard, dry staple foods traditionally consumed in high-risk rural areas [8,9]. Conversely, higher intake of fresh fruit and vegetables, as well as regular tea drinking, has been associated with a lower GC risk [10,11]. Notably, only a minority of heavily exposed individuals eventually develop GC [12], suggesting substantial inter-individual variation in genetic susceptibility.

GC is not a single etiological entity. Anatomically, it is divided into cardia and non-cardia cancers, which differ in time trends and risk-factor profiles [6]. Non-cardia GC has declined in parallel with falling *H. pylori* prevalence, whereas cardia GC has remained stable or increased in many regions, in association with obesity and gastroesophageal reflux [13,14]. Inherited susceptibility also appears to vary by subsite: the 10q23 PLCE1 locus (rs2274223) was initially identified in a GWAS [15] and subsequently confirmed by meta-analyses to be preferentially associated with cardia adenocarcinoma in Asian populations [16,17], whereas the 5p13.1 PRKAA1 locus (rs13361707) and the 3q13.31 ZBTB20 locus (rs9841504) have been specifically linked to non-cardia disease in a Chinese GWAS [18]. These differences support separate analysis of the two subsites.

Because early GC is largely asymptomatic, and endoscopy with biopsy is invasive, costly, and unsuitable for repeated population screening, less invasive biomarkers are needed. The conventional serum markers CEA, CA19-9, CA72-4, and CA125 are inexpensive and minimally invasive but lack adequate sensitivity and specificity [19], especially for early-stage disease. A proteomic study identified four novel serum biomarkers—COPS2, CTSF, NT5E, and TERF1—with substantially higher diagnostic accuracy than conventional markers [20]. However, how these proteins are regulated remains largely unexplored, and whether inherited variation contributes to GC susceptibility is also unclear [21]. It should also be noted that analytical normalization can materially affect the interpretation of candidate cancer biomarkers [22].

MicroRNAs (miRNAs) are approximately 22-nucleotide non-coding RNAs that bind seed sites in the 3′-untranslated region (3′UTR) of target mRNAs, resulting in translational repression or transcript degradation. Approximately half of miRNA genes are located in cancer-associated fragile chromosomal regions, and miRNAs contribute to multiple cancer hallmarks as oncogenes or tumor suppressors [23,24]. A SNP within a 3′UTR miRNA recognition element can alter miRNA binding affinity and thereby modulate target-gene expression [25]; however, the functional consequences of such variants are context-dependent and have been demonstrated experimentally for only a limited number of cases. The let-7 complementary-site variant in the KRAS 3′UTR was the first example, where the variant increased KRAS expression and lung cancer risk [26]. Our candidate selection therefore combined target-site and miRNA-gene variants. Bioinformatic prediction indicated that miR-29b binds the *CTSF* 3′UTR and that the miRNA-gene SNP rs157906 produces a large predicted change in duplex free energy (ΔΔG = 4.4 kcal/mol), whereas miR-30a binds the *NT5E* 3′UTR and its miRNA-gene SNP rs2222722 yields ΔΔG = 2.3 kcal/mol. The target-site SNPs rs4576 (*CTSF* 3′UTR) and rs6913634 (*NT5E* 3′UTR) lie within the predicted miR-29b and miR-30a binding regions, respectively, and were genotyped together with the miRNA-gene variants. Members of the miR-29 and miR-30 families act predominantly as tumor suppressors in the gastric epithelium [27], with miR-29 targeting AKT2 and CDC42 [28,29], and miR-30a inhibiting cell growth and migration in gastric cancer [30].

Complex diseases such as GC are driven by gene–environment interactions, a central premise of nutrigenetics [31]. Single variants often have modest effects, whereas multiple risk alleles combined with specific environmental exposures can substantially elevate risk [32]. In GC, gene–diet interactions are increasingly recognized, such as between polygenic risk scores and coffee [33]. At the miRNA level, although variants in miRNA genes and their binding sites have been systematically evaluated as biomarkers for GC [21], their interaction with dietary exposures remains largely unexplored. Specifically, whether miRNA-related variants in the serum-marker genes *COPS*2, *CTSF*, and *NT5E* interact with diet, and whether this varies by GC subsite, has not been systematically evaluated in independent populations.

We therefore conducted a 1:1 matched case-control study in a high-incidence county in Fujian Province, China, and genotyped miRNA-related SNPs in *COPS*2, *CTSF*, and *NT5E* along with their cognate miRNAs. Conditional logistic regression was applied to evaluate their associations with GC risk by anatomical subsite (overall, cardia, and non-cardia). Furthermore, we employed generalized multifactor dimensionality reduction (GMDR) to identify optimal gene–diet interaction models. We assessed both multiplicative and additive interactions; specifically, the relative excess risk due to interaction (RERI), attributable proportion (AP), and synergy index (S) was calculated to quantify biological synergy on the additive scale. This study aimed to investigate whether these miRNA-related variants are associated with GC risk by subsite and to determine whether dietary exposures modify these associations, thereby providing novel insights into gene–diet interaction in gastric carcinogenesis.

## 2. Materials and Methods

### 2.1. Study Design and Participants

This was a 1:1 individually matched case-control study conducted in a high-GC-incidence county in Fujian Province, China, a region with persistently high GC mortality (crude mortality 54.14 per 100, 000 in the 2011–2012 all-cause mortality survey). GC cases were recruited from the local county hospital between April 2019 and November 2025. Inclusion criteria for cases were: (1) histologically confirmed gastric adenocarcinoma diagnosed by surgery or endoscopy; (2) diagnosis between April 2019 and November 2025; and (3) local residence for at least 10 years. Exclusion criteria were gastric inflammation, benign lesions, or severe illness preventing clear communication. Healthy controls were selected from the same townships or townships with similar GC mortality, using stratified random sampling across high-, medium-, and low-mortality townships based on the previous all-cause mortality survey (2011–2012). Controls were individually matched to cases by sex and age (±3 years), and additionally had no history of cancer, no first-degree-relative relationship with the matched case, and local residence for at least 10 years. Assuming an average exposure prevalence of 0.30, a minimum detectable odds ratio of 1.5, α = 0.05, and β = 0.10, the required sample size was 566 matched pairs; 622 case-control pairs were enrolled in the questionnaire survey. For the present genetic analysis, 550 case-control pairs (97.2% of the required sample size) were included after excluding pairs with missing blood samples, insufficient DNA quality or concentration, or failed genotyping.

### 2.2. Questionnaire and Dietary Assessment

A structured questionnaire (“Health Habits and Lifestyle Questionnaire”), adapted from the national gastric cancer collaborative study questionnaire, was administered face-to-face by trained interviewers (two county-hospital nurses and public-health graduate students from Fujian Medical University). Information collected comprised eight categories (337 items): demographic characteristics (age, sex, height, weight, marital status, education, occupation, and income), medical records, occupational history, dietary habits and food intake, history of gastrointestinal diseases, family history of cancer, and psychosocial factors. Dietary and behavioral variables included hard-food preference, food temperature, eating speed, meal regularity, breakfast skipping, salt taste preference, pickled vegetable intake, tea drinking, fruit and vegetable intake, and post-meal labor; lifestyle variables included smoking (≥1 cigarette/day for ≥6 months), alcohol drinking, and pesticide exposure. For food intake items, participants were classified into three categories: non-consumers (coded 0), and consumers divided at the median intake into low- and high-intake groups (one liang equals 50 g); detailed variable definitions and coding are provided in Table S1. Questionnaire data were double-entered in EpiData 3.1 (EpiData Association, Odense, Denmark) with consistency checks, and 5% of questionnaires were randomly re-checked against the original records, with any discrepancies corrected. All dietary, lifestyle, and psychosocial exposures were ascertained by self-report at the time of the interview. Because interviews for cases were conducted after the histological diagnosis had been confirmed, whereas those for controls were conducted at enrollment, these exposures were recalled retrospectively; participants were asked to report their habitual intake, and several items (for example, monthly household income and daily tea consumption) explicitly referred to the decade preceding enrollment. Potential recall bias arising from this retrospective assessment is discussed in Section 4.7.

### 2.3. Blood Collection and Individual Numbering

Peripheral venous blood (5 mL) was collected from each participant using EDTA anticoagulant tubes(Becton Dickinson, Franklin Lakes, NJ, USA); for cases, blood was drawn before any clinical treatment, and for controls, after the questionnaire interview. Samples were centrifuged at 3000 rpm for 10 min, and plasma, leukocytes, and erythrocytes were separated into aliquots and stored at −80 °C until use. Each participant was assigned a unique individual number, which was used consistently on the questionnaire, blood tubes, and all laboratory records to ensure anonymous linking of the data.

### 2.4. DNA Extraction and Genotyping

Genomic DNA was extracted from peripheral blood leukocytes using DNAzol reagent (Bioteke, Beijing, China). DNA concentration and purity were determined using a NanoDrop ND-1000 spectrophotometer (NanoDrop Technologies, Wilmington, DE, USA). Genotyping was performed by CapitalBio Technology (Beijing, China) using the Sequenom MassARRAY iPLEX platform (Sequenom, San Diego, CA, USA). PCR and single-base-extension primers (Table S3) were designed using MassARRAY Assay Design software (version 3.1; Sequenom, San Diego, CA, USA). The assay combines multiplex PCR, shrimp alkaline phosphatase treatment, single-base extension, resin purification, chip dispensing (MassARRAY Nanodispenser, Sequenom, San Diego, CA, USA), and matrix-assisted laser desorption/ionization time-of-flight mass spectrometry (MALDI-TOF MS; MassARRAY Compact System, Sequenom, San Diego, CA, USA). Genotype calling was performed with TYPER 4.0 software (Sequenom). Laboratory personnel were blinded to case-control status; 2% of samples were randomly selected for blinded duplicate genotyping, and discordant results were resolved by re-genotyping. Samples with failed genotyping were excluded.

### 2.5. SNP Selection

Candidate genes (*COPS2*, *CTSF*, *NT5E*, and *TERF1*) were derived from a previous proteomic study that identified their encoded proteins as novel serum biomarkers for GC [20]. SNPs were selected using the following bioinformatic pipeline: (1) 3′UTR variants of the four genes were retrieved from the NCBI dbSNP database; (2) variants with a minor allele frequency (MAF) > 0.05 in Chinese populations were identified using the 1000 Genomes Project database; (3) miRNAs binding to each 3′UTR were predicted using TargetScan Human 7.1 and microRNA.org; (4) SNPs within the corresponding miRNA genes were retrieved from the miRNASNP database, again requiring MAF > 0.05 in Chinese populations; and (5) for the miRNA-gene variants, RNAcofold(ViennaRNA Package, version 2.4; University of Vienna, Vienna, Austria) was used to calculate the change in duplex Gibbs free energy (ΔΔG, kcal/mol) between wild-type and mutant alleles, and variants with larger ΔΔG values were prioritized. After primer design and assay validation, eight SNPs were successfully genotyped (Table S2): rs1048975 (*COPS2* 3′UTR), rs4576 (*CTSF* 3′UTR), rs6913634 (*NT5E* 3′UTR), rs157906 and rs7781163 (miR-29b), rs2724377 (*miR-29c*), rs2222722 (miR-30a), and rs2829801 (miR-155-5p). The *TERF*1 rs12678406 assay could not be optimized and was therefore not carried forward, so no *TERF*1 variant was analyzed. Because rs2724377 deviated from the Hardy–Weinberg equilibrium (HWE) in controls (χ^2^ = 14.22, *p* < 0.001), it was excluded from downstream regression analyses, leaving seven SNPs in the final analysis.

### 2.6. Statistical Analysis

All statistical analyses were performed using SPSS version 29.0 (IBM Corp., Armonk, NY, USA) and GMDR software v1.0 (Zhejiang University, Hangzhou, China). The significance level was set at two-sided *p* < 0.05. Categorical baseline characteristics were compared between cases and controls using the chi-square test. HWE in controls was tested using the chi-square goodness-of-fit test. Genotype and allele frequencies were compared between cases and controls using the chi-square test for descriptive purposes; the matched design was accounted for in the conditional logistic regression models. Associations of individual SNPs with GC risk were estimated by conditional logistic regression—conditioned on the matched-pair identifier—and adjusted odds ratios (aORs) with 95% confidence intervals (CIs) were calculated under co-dominant, dominant, and recessive models, with adjustment for marital status, education, occupation, and income. Analyses were conducted separately for overall GC, cardia GC, and non-cardia GC. Because multiple SNPs, inheritance models, tumor subsites, and interaction tests were examined and no adjustment for multiple comparisons was prespecified, these analyses are exploratory, and the results should be interpreted as hypothesis-generating.

Gene–gene and gene–environment interactions were explored using generalized multifactor dimensionality reduction (GMDR) [34]. The twelve environmental, lifestyle, and psychosocial factors carried forward from the companion risk-factor survey in the same population (smoking, pesticide exposure, hard-food preference, fast eating, high-salt diet, irregular meals, post-meal labor, pickled vegetable intake, gastric disease history, family history of cancer, type A personality, and proneness to anger) were entered into GMDR together with the seven SNPs that conformed to HWE. Candidate models were restricted to those containing at least one genetic factor, because the aim was to detect gene–environment interaction; among such models, the best model was defined as the one with the highest testing balanced accuracy together with a cross-validation consistency of at least 8/10 and a sign-test *p* < 0.05. For the best gene–environment interaction model, joint effects were analyzed using conditional logistic regression stratified by genotype and exposure. Multiplicative interaction was assessed by including a product term in the logistic regression model. Additive interaction was evaluated using the relative excess risk due to interaction (RERI), attributable proportion (AP), and synergy index (S); 95% CIs were estimated by the delta method, using the asymptotic covariance matrix from SPSS version 29.0 and the Excel spreadsheet developed by Andersson et al. [35]. Additive interaction was considered present when the RERI and AP 95% CIs excluded 0; the synergy index (S) was reported as a supporting measure, because its confidence interval is unstable when the interaction is weak. Because GMDR does not accommodate the matched design, pairing was not retained during interaction screening; matching was preserved only in the conditional logistic regression models.

## 3. Results

### 3.1. Baseline Characteristics

After excluding pairs with missing blood samples, inadequate DNA quality, or failed genotyping, 550 GC cases and 550 matched controls were included. Cases comprised 407 men (74.0%) and 143 women (26.0%), aged 33–93 years (mean 66.34 ± 9.66 years; median 65), and controls 407 men (74.0%) and 143 women (26.0%), aged 35–91 years (mean 65.91 ± 9.87 years; median 66). Sex, age group, marital status, and occupation were balanced between groups (all *p* > 0.05), whereas education (*p* = 0.002) and monthly income 10 years prior to enrollment (*p* < 0.001) differed significantly (Table 1); these two variables were therefore included as covariates in all conditional logistic regression models, together with marital status and occupation.

### 3.2. Hardy–Weinberg Equilibrium

Of the eight genotyped SNPs, seven conformed to Hardy–Weinberg equilibrium (HWE) in controls (all *p* > 0.05), indicating that the genotype distributions were consistent with Hardy–Weinberg expectations (Table S4). The *miR-29c* rs2724377 genotype distribution deviated from HWE (χ^2^ = 14.22, *p* < 0.001) and was excluded from all downstream regression analyses, leaving seven SNPs. Among cases, no substantial deviation from HWE was observed for any of the seven retained SNPs nor for the excluded rs2724377 variant.

### 3.3. Genotype and Allele Distributions

Genotype distributions differed significantly between cases and controls for *CTSF* rs4576 (χ^2^ = 10.35, *p* = 0.006) and *NT5E* rs6913634 (χ^2^ = 9.31, *p* = 0.009); the distributions of the other five SNPs did not differ (all *p* > 0.05) (Table S5). At the allele level, the rs4576 G allele (31.7% vs. 26.9%; χ^2^ = 6.16, *p* = 0.013) and the rs6913634 T allele (8.0% vs. 4.8%; χ^2^ = 9.28, *p* = 0.002) were more frequent in cases than in controls; no significant allele-frequency difference was observed for the remaining SNPs (Table S6).

### 3.4. Associations Between the SNPs and Gastric Cancer Risk

In conditional logistic regression adjusted for marital status, education, occupation, and income, the *CTSF* rs4576 AG genotype (adjusted odds ratio [aOR] = 1.51, 95% CI: 1.16–1.98) and the dominant model AG + GG (aOR = 1.48, 95% CI: 1.14–1.92) were associated with increased overall GC risk. Stratified analysis showed that these associations were confined to non-cardia GC (AG: aOR = 1.57, 95% CI: 1.08–2.29; dominant: aOR = 1.56, 95% CI: 1.09–2.24), with no significant association for cardia GC. The *NT5E* rs6913634 CT genotype (aOR = 1.63, 95% CI: 1.08–2.44) and dominant model CT + TT (aOR = 1.62, 95% CI: 1.09–2.40) increased overall GC risk; in the cardia stratum, the associations were stronger (CT: aOR = 2.38, 95% CI: 1.27–4.45; dominant: aOR = 2.51, 95% CI: 1.35–4.67), whereas no association was found for non-cardia GC. The *miR-30a* rs2222722 AA genotype and its recessive model were associated with cardia GC only (AA vs. GG: aOR = 1.80, 95% CI: 1.00–3.25; recessive: aOR = 1.84, 95% CI: 1.11–3.04). No significant associations were observed for *COPS2* rs1048975, *miR-29b* rs157906 or rs7781163, or *miR-155-5p* rs2829801 under any model (Table 2; complete estimates in Table S7). No *TERF*1 variant could be evaluated because its assay failed optimization (Section 2.5).

### 3.5. GMDR Gene–Gene Interaction Analysis

GMDR was applied to the seven SNPs for overall GC, cardia GC, and non-cardia GC (Table 3A). For overall GC, the three-locus model comprising rs1048975, rs4576, and rs6913634 showed the highest testing balanced accuracy (0.5682) and a cross-validation consistency of 10/10, but the sign test did not reach statistical significance (*p* = 0.0547). No significant gene–gene interaction model was identified in the cardia or non-cardia strata (all sign-test *p* > 0.05). Of note, rs6913634 was consistently retained in cardia models and rs4576 in non-cardia models, in line with the single-locus findings.

### 3.6. GMDR Gene–Environment Interaction Analysis

The twelve environmental, lifestyle, and psychosocial factors carried forward from the companion risk-factor survey were entered into GMDR together with the seven SNPs (Table 3B). For overall GC, the three-factor model consisting of hard-food preference, pickled vegetable intake, and *CTSF* rs4576 was selected as the best model, with the highest testing balanced accuracy (0.6845), a cross-validation consistency of 10/10, and a significant sign test (*p* = 0.0010). The same three-factor model was selected for cardia GC (testing balanced accuracy 0.6720; cross-validation consistency 8/10; *p* = 0.0010) and was carried forward to interaction analysis. For cardia GC, the environment-only model consisting of hard-food preference alone had a numerically higher testing balanced accuracy (0.6806) and a cross-validation consistency of 10/10; because this model contains no genetic factor, it cannot address gene–environment interaction and was therefore not eligible under the predefined selection rule. Accordingly, the cardia model should be regarded as hypothesis-generating. For non-cardia GC, the three-factor model was unstable (cross-validation consistency 5/10), so no reliable gene–environment interaction model could be established.

### 3.7. Joint Effects and Interaction Analyses

For overall GC, under the rs4576 dominant model, G-allele carriers who reported a preference for hard food had a 5.75-fold higher risk than AA carriers without this preference (aOR = 5.75, 95% CI: 3.78–8.76), and G-allele carriers consuming pickled vegetables had a 4.44-fold increased risk compared with AA carriers who did not (aOR = 4.44, 95% CI: 2.92–6.74) (Table 4). No significant multiplicative interaction was detected (hard food: product-term aOR = 1.26, 95% CI: 0.70–2.25; pickled vegetables: aOR = 0.77, 95% CI: 0.44–1.36). In contrast, evidence of a positive additive interaction was observed between the rs4576 dominant model and hard-food preference (RERI = 1.65, 95% CI: 0.049–3.26; AP = 0.29, 95% CI: 0.053–0.52), indicating that approximately 29% of the joint effect was attributable to the interaction; the corresponding synergy index was 1.53 (95% CI: 1.00–2.35), consistent in direction but borderline, with the lower limits of the RERI (0.049) and AP (0.053) intervals lying close to the null; no additive interaction was observed between the dominant model and pickled vegetable intake or for the recessive model with either factor (Table 5). In the cardia stratum, joint risks were similarly elevated (G allele plus hard food: aOR = 7.48, 95% CI: 3.83–14.63; G allele plus pickled vegetables: aOR = 3.60, 95% CI: 1.98–6.53), but neither multiplicative nor additive interaction reached statistical significance (all RERI 95% CIs included 0 and all S 95% CIs included 1) (Table 4 and Table 5; recessive-model estimates in Table S8).

## 4. Discussion

Gastric cancer (GC) is a multistep, multifactorial disease driven by genetic and environmental factors, yet only a minority of heavily exposed individuals develop it, indicating marked inter-individual variation in susceptibility [14,36]. miRNA-related single-nucleotide polymorphisms (SNPs) in *COPS*2, *CTSF*, and *NT5E—*genes encoding recently identified serum biomarkers of GC—and in their cognate miRNA genes have rarely been examined for GC susceptibility [20]. We therefore conducted a 1:1 matched case-control study of 550 pairs in a high-incidence county of Fujian Province, China, using conditional logistic regression for main effects and generalized multifactor dimensionality reduction (GMDR) combined with crossover and additive interaction measures for gene–diet interactions. *CTSF* rs4576, *NT5E* rs6913634, and miR-30a rs2222722 were associated with GC risk, with distinct anatomical-site preferences (rs4576 for non-cardia GC; rs6913634 and rs2222722 for cardia GC). GMDR identified hard-food preference, pickled vegetable intake, and rs4576 as the optimal three-factor model, and crossover analysis indicated a positive additive interaction between the rs4576 dominant model and hard-food preference. This study provides novel epidemiological evidence linking miRNA-related polymorphisms in *CTSF* and *NT5E* to GC risk in this high-risk Chinese population, and highlights a specific gene–diet interaction at the *CTSF* locus.

### 4.1. CTSF rs4576 and Non-Cardia Gastric Cancer

*CTSF* encodes cathepsin F, a papain-family lysosomal cysteine protease that mediates intracellular protein degradation. *CTSF* has been identified as a breast cancer risk gene, with genetically predicted higher *CTSF* levels associated with an increased risk of breast cancer [37]. The miR-29b gene variant rs157906 was predicted to produce the largest change in duplex free energy (ΔΔG = 4.4 kcal/mol), implicating altered miR-29b function. In addition, rs4576 lies within the predicted miR-29b binding region of the *CTSF* 3′-UTR, so this substitution may also affect miR-29b-*CTSF* duplex stability, although no ΔΔG value was computed for this target-site variant. In GC, *miR-29b* is downregulated and acts as a tumor suppressor through *AKT2*-related pathways, and the miR-29 family can activate p53 by repressing *CDC42* and the p85α regulatory subunit of PI3K [28]. A plausible mechanism is therefore that the G allele weakens or abolishes *miR-29b*-mediated post-transcriptional repression, leading to altered *CTSF* expression. Although *CTSF* has been reported to inhibit gastric cancer progression in some contexts [38], cysteine cathepsins as a family have been implicated in tumor angiogenesis, and *CTSF* itself has been functionally linked to the migratory capacity of cancer cells, suggesting that its role may be context-dependent [39]. Consistent with this hypothesis, the AG genotype and the dominant AG + GG model conferred 1.51- and 1.48-fold increased overall GC risks, respectively, and stratified analysis showed that these associations were confined to non-cardia GC (aOR = 1.57 and 1.56). Although functional experiments are required to demonstrate allele-specific miRNA binding, the concordance among the bioinformatic prediction, the direction of the genetic associations, and the established tumor-suppressive function of *miR-29b* identifies *CTSF* rs4576 as a candidate susceptibility variant for non-cardia GC that warrants functional validation.

### 4.2. The NT5E-miR-30a Axis and Cardia Gastric Cancer

*NT5E* encodes ecto-5′-nucleotidase (CD73), a glycosylphosphatidylinositol-anchored membrane glycoprotein that catalyzes the terminal hydrolysis of extracellular ATP to adenosine and functions as a master switch of extracellular nucleotide metabolism. NT5E/CD73 activity is elevated in the tissues and serum of patients with colorectal and other gastrointestinal malignancies and correlates with tumor stage, invasive depth, metastasis, and shorter survival [40]; adenosine accumulation also promotes progression of aggressive breast cancer subtypes [41]. The miR-30a variant rs2222722 was predicted to change duplex free energy by 2.3 kcal/mol, whereas rs6913634 lies within the predicted miR-30a binding region of the *NT5E* 3′-UTR, although no ΔΔG value was computed for this target-site variant. In gastrointestinal cancer studies, *miR-30a-5p* is under-expressed and acts as a tumor suppressor, inhibiting cell growth and migration in gastric cancer [30]. The CT genotype and dominant CT + TT model were associated with overall GC risk (aOR = 1.63 and 1.62), with stronger associations restricted to the cardia stratum (aOR = 2.38 and 2.51; T-allele aOR = 1.59). Importantly, the AA genotype of *miR-30a* rs2222722—the miRNA predicted to regulate *NT5E*—was independently associated with cardia GC (AA vs. GG aOR = 1.80; recessive-model aOR = 1.84). The convergence of a 3′-UTR variant in the host gene and a variant in its regulatory miRNA on the same anatomical subtype strengthens the candidacy of the *NT5E–miR-30a* axis in cardia carcinogenesis, although the molecular consequences of each variant remain to be experimentally defined.

### 4.3. Etiologic Heterogeneity Between Cardia and Non-Cardia Gastric Cancer

Cardia (proximal) and non-cardia (distal) GC differ in their descriptive epidemiology and etiology: cardia tumors are principally linked to Barrett’s esophagus, gastroesophageal reflux, and obesity and are less closely related to *H. pylori* infection, whereas non-cardia tumors are strongly *H. pylori*-dependent [14]; genetic susceptibility loci likewise show subsite specificity, as illustrated by the 10q23 variant rs2274223 for cardia GC [42] and by non-cardia-specific susceptibility loci identified in Asian GWAS [43]. Our results add molecular evidence for this heterogeneity. First, rs4576 was associated only with non-cardia GC, whereas rs6913634 and rs2222722 were associated only with cardia GC. Second, GMDR consistently retained rs6913634 in cardia gene–gene models and rs4576 in non-cardia models. These parallel observations support the treatment of cardia and non-cardia GC as distinct entities in future genetic and gene–environment studies rather than pooling all gastric tumors.

### 4.4. Negative Loci and Unexamined Variants

Several plausible loci were null. *COPS2* rs1048975 showed no association under co-dominant, dominant, or recessive models at any subsite, despite predictions that this 3′-UTR region binds *miR-497* and *miR-15a-3p*. *COPS2* (CSN2) has context-dependent tumor-suppressive and oncogenic properties, and its partial loss can confer resistance to p53- and p16INK4a-induced growth arrest [44]; a single tagging variant may fail to capture the relevant biology, and limited statistical power cannot be excluded. Similarly, the *miR-29b* rs157906, *miR-29b* rs7781163, and *miR-155-5p* rs2829801 variants were not associated with GC at any subsite, and *miR-29c* rs2724377 was removed after deviation from the Hardy–Weinberg equilibrium, leaving its role untested. The *TERF1* rs12678406 primer assay could not be optimized, so this locus could not be evaluated at all. These negative findings should be interpreted as locus-specific rather than pathway-level evidence: only a small, bioinformatics-selected set of variants was genotyped, and these genes are subject to many other layers of regulation.

### 4.5. Gene–Gene Interaction

GMDR did not identify a statistically significant gene–gene interaction. For overall GC, the three-locus model rs1048975–rs4576–rs6913634 had the highest testing balanced accuracy (0.5682) and a cross-validation consistency of 10/10, but the sign test fell just short of significance (*p* = 0.0547), and no model in either tumor stratum was significant. We therefore refrain from claiming epistasis. With seven examined loci and a few hundred informative matched pairs per stratum, the study had limited power for high-order interactions; the fact that the borderline model combined the two individually significant loci suggests it may be enriched for genuine effects and warrants replication in larger samples.

### 4.6. Gene–Diet Interaction and Public-Health Implications

The gene–environment analysis focused on dietary and lifestyle factors carried forward from the companion risk-factor survey in the same population. GMDR selected hard-food preference and pickled vegetable intake together with rs4576 as the best model for overall GC (testing balanced accuracy 0.6845; cross-validation consistency 10/10; *p* = 0.0010), and the same three-factor model was selected for cardia GC under the predefined selection rule. On the additive scale, evidence was found for a positive additive interaction between the rs4576 dominant model and hard-food preference (RERI = 1.65, 95% CI 0.049–3.26; AP = 0.29, 95% CI 0.053–0.52; S = 1.53, 95% CI 1.00–2.35): the combined excess risk exceeded the sum of the individual effects, and approximately 29% of the joint effect was attributable to the interaction. The synergy index was consistent in direction but borderline (whose lower confidence limit rounds to 1.00 at two decimal places), and this estimate should be regarded as exploratory. No multiplicative interaction was evident, which is not surprising because biological synergy is often detectable on the additive rather than the multiplicative scale. Mechanistically, long-term consumption of hard-textured food can produce repeated mechanical injury, erosion, and congestion of the gastric mucosa and promote precancerous lesions, whereas pickled vegetables deliver high salt and *N-*nitroso compounds [45]; the rs4576 G allele, plausibly through de-repression of *CTSF*, may amplify the cellular response to these dietary insults. This interpretation accords with earlier evidence that gastric susceptibility variants interact with environmental exposures such as *H. pylori* infection [46].

These joint-risk estimates are exploratory and should be interpreted as hypothesis-generating rather than as a basis for genotype-guided dietary advice. G-allele carriers preferring hard food had a 5.75-fold increased risk, and G-allele carriers consuming pickled vegetables a 4.44-fold increased risk, relative to AA carriers without the exposure; the corresponding cardia estimates were even higher (7.48 and 3.60). Although formal interaction did not reach significance in the smaller cardia stratum, and no stable gene–environment model could be established for non-cardia GC (cross-validation consistency 5/10), effect directions were consistent across analyses and subsites. Because inherited susceptibility cannot presently be modified, reducing intake of hard-textured foods and pickled vegetables—measures that are advisable in this population irrespective of genotype—may be of particular relevance among carriers of the rs4576 risk genotype; however, this exploratory finding requires replication in independent populations, and it does not yet support genotype-targeted dietary recommendations.

### 4.7. Strengths and Limitations

Strengths include the 1:1 individually matched design, with 550 case-control pairs from one large-scale epidemiological survey in a high-GC-incidence county; matched conditional logistic regression that preserved the pairing in all regression-based analyses; parallel interrogation of host-gene 3′-UTR variants and their cognate miRNA variants; a systematic interaction workflow combining GMDR, crossover analysis, and both multiplicative and additive interaction metrics (RERI, AP, and S); and stratified analysis by tumor subsite. Several limitations should be acknowledged. First, hemolysis of part of the collected blood samples compromised DNA quality and led to exclusion of affected pairs, which could introduce selection bias. Second, the functional consequences of rs4576, rs6913634, and rs2222722 were inferred from bioinformatic predictions alone; allele-specific reporter assays and expression studies are needed before causal mechanisms can be claimed. Third, statistical power was limited for rare homozygous genotypes, subsite-specific estimates, and high-order interactions, so null results do not exclude small effects. Fourth, dietary exposures were self-reported through face-to-face interview and are susceptible to recall and misclassification errors. Fifth, GMDR does not accommodate the matched design, so pairing was not retained during interaction screening and matching was preserved only in the conditional logistic regression models. No adjustment for multiple comparisons was applied, and all analyses should therefore be regarded as exploratory. *H. pylori* infection, a major risk factor for GC in this population, was not included in the present interaction models, so residual confounding by *H. pylori* cannot be excluded. Other dietary and lifestyle exposures that were not entered into the interaction models—for example, salt intake, tea drinking, and fruit and vegetable consumption—may likewise confound or modify the reported associations, and residual confounding by these correlated exposures cannot be ruled out. In addition, dietary and lifestyle exposures were reported after diagnosis in cases and at enrollment in controls, and several questionnaire items (including household income) referred to the decade preceding diagnosis, so differential recall between cases and controls may have biased the interaction estimates. Finally, participants came from a single county in Southeast China, and multi-center replication in larger populations of diverse ancestry is required.

## 5. Conclusions

This study provides preliminary evidence that miRNA-related polymorphisms in *CTSF* and *NT5E*, and in miR-30a, a putative regulator of *NT5E*, are associated with GC susceptibility in a Chinese high-incidence population. We observed different association patterns between cardia and non-cardia disease (non-cardia for *CTSF* rs4576; cardia for *NT5E* rs6913634 and miR-30a rs2222722). However, since we did not perform formal statistical comparisons between these two subtypes, these findings should be viewed as descriptive observations rather than confirmed subtype-specific differences. The rs4576 dominant model also showed a positive additive interaction with hard-food preference, and carriers of the risk allele who consumed hard-textured or pickled foods had markedly higher joint risks. Overall, these findings support a gene–diet interaction model in gastric carcinogenesis and suggest that diet may be particularly relevant for individuals with genetic susceptibility. However, as this was an exploratory study, further functional experiments and validation in independent populations are needed before making any genotype-specific dietary recommendations.

## Figures and Tables

**Table 1:** Demographic characteristics of gastric cancer cases and matched controls.

Characteristic	Cases (*n* = 550), *n* (%)	Controls (*n* = 550), *n* (%)	χ^2^	*p*
Sex			0.00	1.00
Male	407 (74.0)	407 (74.0)		
Female	143 (26.0)	143 (26.0)		
Age group (years)			3.42	0.49
≤45	11 (2.0)	10 (1.8)		
46–55	49 (8.9)	64 (11.6)		
56–65	216 (39.3)	199 (36.2)		
66–75	179 (32.5)	190 (34.5)		
≥76	95 (17.3)	87 (15.8)		
Age (years), mean ± SD	66.34 ± 9.66	65.91 ± 9.87	—	—
Education			12.15	0.002
Primary school or below	432 (78.5)	382 (69.5)		
Junior high	75 (13.6)	101 (18.4)		
Senior high or above	43 (7.8)	67 (12.2)		
Marital status			0.32	0.85
Married	504 (91.6)	499 (90.7)		
Widowed	42 (7.6)	46 (8.4)		
Other	4 (0.7)	5 (0.9)		
Occupation			0.29	0.59
Farmer	399 (72.5)	391 (71.1)		
Other	151 (27.5)	159 (28.9)		
Monthly income 10 years prior (CNY)			65.00	<0.001
>600	136 (24.7)	193 (35.1)		
300–600	174 (31.6)	242 (44.0)		
<300	240 (43.6)	115 (20.9)		

SD, standard deviation; CNY, Chinese yuan. Age groups were compared by the χ^2^ test; continuous age is presented descriptively as mean ± SD.

**Table 2:** Significant associations between the miRNA-related SNPs and gastric cancer risk (conditional logistic regression, adjusted for marital status, education, occupation, and income).

SNP (Gene)	Comparison (Model)	Overall GC, aOR (95% CI)	Cardia GC, aOR (95% CI)	Non-Cardia GC, aOR (95% CI)
rs4576 (*CTSF*)	AG vs. AA (co-dominant)	1.51 (1.16–1.98)	1.44 (0.97–2.13)	1.57 (1.08–2.29)
	AG + GG vs. AA (dominant)	1.48 (1.14–1.92)	1.39 (0.95–2.03)	1.56 (1.09–2.24)
	GG vs. AA + AG (recessive)	1.05 (0.64–1.73)	0.89 (0.40–2.00)	1.19 (0.62–2.58)
rs6913634 (*NT5E*)	CT vs. CC (co-dominant)	1.63 (1.08–2.44)	2.38 (1.27–4.45)	1.28 (0.74–2.21)
	CT + TT vs. CC (dominant)	1.62 (1.09–2.40)	2.51 (1.35–4.67)	1.24 (0.72–2.11)
	TT vs. CC + CT (recessive)	1.34 (0.28–6.38)	— ^a^	0.77 (0.13–4.60)
rs2222722 (*miR-30a*)	AA vs. GG (co-dominant)	1.29 (0.89–1.87)	1.80 (1.00–3.25)	1.00 (0.61–1.65)
	GA + AA vs. GG (dominant)	1.09 (0.83–1.45)	1.16 (0.74–1.80)	1.05 (0.72–1.53)
	AA vs. GG + GA (recessive)	1.27 (0.92–1.73)	1.84 (1.11–3.04)	0.96 (0.63–1.47)

^a^ Not estimable: 2 TT cases and 0 TT controls in the cardia stratum. aOR, adjusted odds ratio; CI, confidence interval. Estimates for rs1048975, rs157906, rs7781163, and rs2829801 were all non-significant and are shown in Table S7.

**Table 3:** Best GMDR models for gene–gene (**A**) and gene–environment (**B**) interactions.

Outcome	Best Models	Training Balanced Accuracy	Testing Balanced Accuracy	Sign Test *p*	CV Consistency
(**A**) Gene–gene					
Overall GC	S14 (rs4576)	0.5473	0.5473	0.0547	10/10
	S14 S15 (rs4576, rs6913634)	0.5595	0.5336	0.3770	6/10
	S13 S14 S15 (rs1048975, rs4576, rs6913634)	0.5738	0.5682	0.0547	10/10
Cardia GC	S15 (rs6913634)	0.5520	0.4998	0.1719	7/10
	S15 S18 (rs6913634, rs2222722)	0.5711	0.5129	0.6230	6/10
	S16 S18 S19 (rs157906, rs2222722, rs2829801)	0.5843	0.4914	0.6230	4/10
Non-cardia GC	S14 (rs4576)	0.5510	0.5508	0.3770	10/10
	S14 S15 (rs4576, rs6913634)	0.5667	0.5081	0.3770	5/10
	S13 S14 S15 (rs1048975, rs4576, rs6913634)	0.5756	0.5144	0.6230	7/10
(**B**) Gene–environment					
Overall GC	S3 (hard-food preference)	0.6664	0.6664	0.0010	10/10
	S3 S4 (hard food, fast eating)	0.6709	0.6664	0.0010	10/10
	S3 S8 S14 (hard food, pickled vegetables, rs4576)	0.6845	0.6845	0.0010	10/10
Cardia GC	S3 (hard-food preference)	0.6801	0.6806	0.0010	10/10
	S3 S8 (hard food, pickled vegetables)	0.6801	0.6806	0.0010	8/10
	S3 S8 S14 (hard food, pickled vegetables, rs4576)	0.6912	0.6720	0.0010	8/10
Non-cardia GC	S3 (hard-food preference)	0.6560	0.6559	0.0010	10/10
	S3 S8 (hard food, pickled vegetables)	0.6624	0.6622	0.0010	10/10
	S3 S8 S14 (hard food, pickled vegetables, rs4576)	0.6826	0.6544	0.0010	5/10

GMDR, generalized multifactor dimensionality reduction; CV, cross-validation. Environmental factors: S1 smoking, S2 pesticide exposure, S3 hard-food preference, S4 fast eating, S5 high-salt diet, S6 irregular meals, S7 post-meal labor, S8 pickled vegetable intake, S9 gastric disease history, S10 family history of cancer, S11 type A personality, S12 proneness to anger; genetic factors: S13 rs1048975, S14 rs4576, S15 rs6913634, S16 rs157906, S17 rs7781163, S18 rs2222722, S19 rs2829801. The S3-S8-S14 model was selected for downstream interaction analysis for overall GC and cardia GC; no stable model was obtained for non-cardia GC.

**Table 4:** Joint effects and multiplicative interaction of the rs4576 dominant model with hard-food preference and pickled vegetable intake for overall and cardia gastric cancer.

Outcome	Dietary Factor	rs4576 Genotype	Exposure	Cases (*n*)	Controls (*n*)	aOR (95% CI)
Overall GC	Hard food	AA	No	60	165	1 (Ref.)
		AG + GG	No	73	151	1.16 (0.73–1.85)
		AA	Yes	178	125	3.94 (2.58–6.02)
		AG + GG	Yes	239	109	5.75 (3.78–8.76)
	Dominant model × hard food					1.26 (0.70–2.25)
	Pickled vegetables	AA	No	79	172	1 (Ref.)
		AG + GG	No	117	146	1.71 (1.13–2.59)
		AA	Yes	159	118	3.35 (2.19–5.12)
		AG + GG	Yes	195	114	4.44 (2.92–6.74)
	Dominant model × pickled vegetables					0.77 (0.44–1.36)
Cardia GC	Hard food	AA	No	29	77	1 (Ref.)
		AG + GG	No	35	72	1.16 (0.60–2.22)
		AA	Yes	74	46	4.67 (2.40–9.09)
		AG + GG	Yes	98	41	7.48 (3.83–14.63)
	Dominant model × hard food					1.39 (0.56–3.43)
	Pickled vegetables	AA	No	35	74	1 (Ref.)
		AG + GG	No	52	61	1.79 (0.98–3.23)
		AA	Yes	68	49	3.47 (1.83–6.58)
		AG + GG	Yes	81	52	3.60 (1.98–6.53)
	Dominant model × pickled vegetables					0.58 (0.25–1.35)

aORs were estimated by crossover analysis, with conditional logistic regression adjusted for marital status, education, occupation, and income. Product-term aORs assess multiplicative interaction. Recessive-model estimates are provided in Table S8.

**Table 5:** Additive interaction of rs4576 with hard-food preference and pickled vegetable intake.

Outcome	rs4576 Model	Dietary Factor	RERI (95% CI)	AP (95% CI)	S (95% CI)
Overall GC	Dominant (AG + GG vs. AA)	Hard food	1.65 (0.049–3.26)	0.29 (0.053–0.52)	1.53 (1.00–2.35)
	Dominant	Pickled vegetables	0.38 (−1.01–1.76)	0.085 (−0.22–0.39)	1.12 (0.73–1.73)
	Recessive (GG vs. AA + AG)	Hard food	−0.95 (−4.09–2.20)	−0.21 (−1.04–0.62)	0.78 (0.33–1.88)
	Recessive	Pickled vegetables	0.79 (−1.39–2.96)	0.24 (−0.29–0.77)	1.53 (0.55–4.28)
Cardia GC	Dominant	Hard food	2.67 (−0.69–6.02)	0.36 (0.015–0.70)	1.70 (0.89–3.26)
	Dominant	Pickled vegetables	−0.65 (−1.61–0.31)	−0.18 (−0.49–0.13)	0.80 (0.55–1.16)
	Recessive	Hard food	−0.84 (−5.43–3.75)	−0.19 (−1.37–1.00)	0.81 (0.23–2.89)
	Recessive	Pickled vegetables	−0.81 (−2.75–1.14)	−0.48 (−1.98–1.01)	0.45 (0.043–4.71)

RERI, relative excess risk due to interaction; AP, attributable proportion; S, synergy index. 95% CIs were estimated by the delta method. Additive interaction was considered present when the RERI and AP CIs excluded 0; the lower limit of the S CI rounds to 1.00 at two decimal places; S is reported as a supporting measure only. Only the dominant-model × hard-food combination for overall GC met the RERI/AP criterion.

## Data Availability

The data that support the findings of this study contain potentially identifying participant information and are not publicly available under the terms of the ethical approval for this study. All summary estimates underlying the reported results are presented in the article and its Supplementary Materials. Requests for data access may be directed to the corresponding author and will be reviewed case by case by the Ethics Committee of Fujian Medical University.

## Supplementary Materials

The following supporting information can be downloaded at: https://www.mdpi.com/article/10.3390/nu18213574/s1.

## Abbreviations

Abbreviation	Full Name
3′UTR	3′-untranslated region
AEG-1	astrocyte elevated gene-1
aOR	adjusted odds ratio
AP	attributable proportion
ASIR	age-standardized incidence rate
ASMR	age-standardized mortality rate
ATP	adenosine triphosphate
CA125	carbohydrate antigen 125
CA19-9	carbohydrate antigen 19-9
CA72-4	carbohydrate antigen 72-4
CDC42	cell division cycle 42
CD73	cluster of differentiation 73
CEA	carcinoembryonic antigen
CI	confidence interval
CNY	Chinese yuan
*COPS*2	COP9 signalosome complex subunit 2
*CTSF*	cathepsin F
DNA	deoxyribonucleic acid
EDTA	ethylenediaminetetraacetic acid
GC	gastric cancer
GMDR	generalized multifactor dimensionality reduction
GWAS	genome-wide association study
*H. pylori*	*Helicobacter pylori*
HWE	Hardy–Weinberg equilibrium
IARC	International Agency for Research on Cancer
KRAS	Kirsten rat sarcoma viral oncogene homolog
MAF	minor allele frequency
MALDI-TOF MS	matrix-assisted laser desorption/ionization time-of-flight mass spectrometry
miRNA	microRNA
mRNA	messenger RNA
MUC5AC	mucin 5AC
*NT5E*	ecto-5′-nucleotidase
OR	odds ratio
p16INK4a	cyclin-dependent kinase inhibitor 2A
p53	tumor protein p53
p85α	phosphatidylinositol 3-kinase regulatory subunit alpha
PCR	polymerase chain reaction
PI3K	phosphoinositide 3-kinase
PLCE1	phospholipase C epsilon 1
PRKAA1	protein kinase AMP-activated catalytic subunit alpha 1
RERI	relative excess risk due to interaction
RNA	ribonucleic acid
S	synergy index
SD	standard deviation
SNP	single nucleotide polymorphism
SPSS	Statistical Package for the Social Sciences
*TERF*1	telomeric repeat binding factor 1
XIAP	X-linked inhibitor of apoptosis
ZBTB20	zinc finger and BTB domain containing 20
