# Supplementary material for: Gene–Nutrition Interaction in Gastric Cancer: miRNA-Related Polymorphisms in CTSF and NT5E, Dietary Exposures, and Site-Specific Risk
Source: Nutrients. 2026 Oct 29;18(21):3574. doi: 10.3390/nu18213574 (PMC13765215; doi:10.3390/nu18213574)
Supplement: Supplementary file 1 [file nutrients-18-03574-s001.zip › nutrients-4620703-supplementary.pdf]

Supplementary Materials

Table S1. Definitions and coding of selected questionnaire variables.

Variable	Categories and coding
Education	Senior high or above = 1; junior high = 2; primary school or below = 3
Marital status	Married = 1; widowed or divorced = 2; unmarried = 3
Occupation	Farmer = 1; other = 0
Average monthly income 10 years prior (CNY)	<300 = 1; 300–600 = 2; >600 = 3
BMI (kg/m²)	Normal (18.5–24) = 1; underweight (<18.5) = 2; overweight (>24) = 3
Refrigerator use (years)	Never = 0; 1–5 = 1; 6–10 = 2; >10 = 3
Smoking	No = 0; yes = 1 (≥1 cigarette/day for ≥6 months)
Smoking (pack-years)	No = 0; <20 = 1; ≥20 = 2 (pack-years = cigarettes/day ÷ 20 × years of smoking)
Tea drinking	No = 0; yes = 1 (≥1 cup/week for ≥6 months)
Age started tea drinking (years)	No = 0; >30 = 1; ≤30 = 2
Tea-drinking duration (years)	No = 0; <25 = 1; ≥25 = 2
Tea type	No = 0; green tea = 1; black tea = 2; Tieguanyin = 3; other = 4
Daily tea intake (cups, 10 years prior)	No = 0; ≤5 = 1; >5 = 2
Monthly tea leaves consumed (liang)	No = 0; ≤10 = 1; >10 = 2
Pesticide exposure	No = 0; yes = 1
Pesticide use (years)	No = 0; <35 = 1; ≥35 = 2
Pesticide amount (jin) a	No = 0; <300 jin = 1; ≥300 jin = 2 (annual amount × years of use)
Food temperature	Relatively cool = 1; relatively hot = 2
Eating speed	Relatively slow = 1; relatively fast = 2
Food hardness	Relatively soft = 1; relatively hard = 2
Meal regularity	Mostly regular = 1; mostly irregular = 2
Breakfast	Usually eats = 1; frequently skips = 2
Salt taste preference	Bland = 1; salty = 2
Spicy food	Occasionally or never = 1; frequently = 2
Sour food	Occasionally or never = 1; frequently = 2
Binge eating	Occasionally or never = 1; frequently = 2
Post-meal labor	Rarely or occasionally = 1; frequently = 2
Red meat (beef, mutton; liang/year)	No = 0; <24 = 1; ≥24 = 2
Fresh eggs (per week)	No = 0; ≤3 = 1; >3 = 2
Fresh fish and shrimp (liang/week)	No = 0; <4 = 1; ≥4 = 2
Shellfish (liang/week)	No = 0; <4 = 1; ≥4 = 2
Kelp and laver (liang/week)	No = 0; <2 = 1; ≥2 = 2
Dairy products (liang/week)	No = 0; <9 = 1; ≥9 = 2
Soy products (liang/week)	No = 0; <4 = 1; ≥4 = 2

Fruit (liang/week)	No = 0; <6 = 1; ≥6 = 2
Vegetables (liang/week)	No = 0; <42 = 1; ≥42 = 2
Edible fungi (liang/week)	No = 0; <2 = 1; ≥2 = 2
Pickled vegetables (liang/week)	No = 0; <6 liang/week = 1; ≥6 liang/week = 2
Garlic (times/week)	<7 = 1; ≥7 = 2
Onion and scallion (times/week)	<7 = 1; ≥7 = 2
Chronic atrophic gastritis	No = 0; yes = 1
Family history of cancer	No = 0; yes = 1
Personality type	Intermediate = 1; type B = 2; type A = 3
Mood	Happy = 1; occasional distress = 2; frequent distress = 3
Proneness to anger	No = 0; yes = 1

a Pesticide amount = annual amount used × years of use; one liang equals 50 g, one jin equals 500 g. The complete questionnaire comprised 337 items across eight categories; only variables relevant to the present analysis are shown.

Table S2. Characteristics of the eight genotyped SNPs.

**Gene**	**miRNA**	**SNP ID**	**Location**	**Alleles**	**MAF (Chinese)**	**ΔΔG (kcal/mol) [b]**
COPS2	—	rs1048975	3′UTR	G/T	0.432	—
CTSF	—	rs4576	3′UTR	A/G	0.277	—
NT5E	—	rs6913634	3′UTR	C/T	0.052	—
CTSF	*miR-29b*	rs157906	miRNA gene	G/A	0.199	4.4
CTSF	*miR-29b*	rs7781163	miRNA gene	T/C	0.214	—
CTSF	*miR-29c*	rs2724377 c	miRNA gene	A/G	0.194	3.4
NT5E	*miR-30a*	rs2222722	miRNA gene	G/A	0.427	2.3
TERF1	*miR-155-5p*	rs2829801	miRNA gene	T/G	0.186	3.3

[b] ΔΔG, change in duplex Gibbs free energy between wild-type and mutant alleles predicted by RNAcofold; —, not applicable (ΔΔG was calculated only for miRNA-gene SNPs; 3′UTR target-site SNPs were genotyped by MAF screening only) or not reported. MAF values were obtained from the 1000 Genomes Project database for Chinese populations. c rs2724377 deviated from Hardy–Weinberg equilibrium in controls ($\chi^2 = 14.22$, $P < 0.001$) and was excluded from downstream regression analyses.

Table S3. PCR amplification and single-base-extension primers for the Sequenom MassARRAY assays.

SNP ID	PCR primer (5′→3′)	Single-base-extension primer (5′→3′)
rs1048975	F: ACGTTGGATGAGAGCAAGAGTGAGTAATGG<br>R: ACGTTGGATGTTGGCTGCCACAAGTAGATG	agggGTAATGGAAATCCAGCTCT
rs4576	F: ACGTTGGATGTGGTTACAAGTTTCCTGGAC<br>R: ACGTTGGATGCGCTTTCCTCTGTTTGATAC	GTTTCCTGGACATGGAG
rs6913634	F: ACGTTGGATGCTATAAGGAGAAAGCCAACT<br>R: ACGTTGGATGGAAATGGATATGAAGAAGAA	ggcgAAATTTCATTAAAATTTGGACAC
rs157906	F: ACGTTGGATGCCTAAATCCATACTATCTA<br>R: ACGTTGGATGCAGCAAAAAATATGTATCTA	cTCCATACTATCTAAATTCAATAACT
rs7781163	F: ACGTTGGATGGGATCCAAAGGATTACTGAG<br>R: ACGTTGGATGTGTGACTTACTTGGCCTCTG	GGCCTCTGAGCTAGTCT
rs2724377	F: ACGTTGGATGGAAGTAGGCAAGATCTGCAC<br>R: ACGTTGGATGCATGTCTGTGTTTTTCATC	TTTCAGGACCCACTTCTTATC
rs2222722	F: ACGTTGGATGGCAAAAGTGACCAAACACAG<br>R:	TTGATGTTAAATGTACGCTTTA

	ACGTTGGATGGTCTAAGTTCACTCAACTGC	
rs2829801	F: ACGTTGGATGACCACTAATTCCCACTCAGG<br>R: ACGTTGGATGTCCAGTGTTTTTGACTGGGC	ctaaTGGGCTAAGCAACCA

Primers were designed using MassARRAY Assay Design software (Sequenom). F, forward; R, reverse.

Table S4. Hardy–Weinberg equilibrium tests for the eight genotyped SNPs in controls.

Gene	SNP	Genotype (observed)	Observed	Expected	χ^2	P
COPS2	rs1048975	GG / GT / TT	169 / 288 / 93	178 / 270 / 102	2.52	0.11
CTSF	rs4576	AA / AG / GG	290 / 224 / 36	294 / 216 / 40	0.69	0.41
NT5E	rs6913634	CC / CT / TT	500 / 47 / 3	498 / 50 / 1	2.57	0.11
miR-29b	rs157906	GG / GA / AA	394 / 138 / 18	390 / 146 / 14	1.84	0.17
miR-29b	rs7781163	TT / TC / CC	400 / 139 / 11	401 / 137 / 12	0.07	0.79
miR-29c	rs2724377	AA / AG / GG	337 / 205 / 8	351 / 177 / 22	14.22	<0.001
miR-30a	rs2222722	GG / GA / AA	150 / 293 / 107	160 / 273 / 117	2.85	0.09
miR-155-5p	rs2829801	TT / TG / GG	338 / 195 / 17	345 / 181 / 24	3.13	0.08

rs2724377 deviated from HWE and was excluded from downstream regression analyses.

Table S5. Genotype distributions of the seven analyzed SNPs in cases and controls.

SNP (gene)	Genotype	Cases, n (%)	Controls, n (%)	χ²	P
rs1048975 (*COPS2*)	GG	150 (27.3)	169 (30.7)	1.61	0.45
	GT	301 (54.7)	288 (52.4)		
	TT	99 (18.0)	93 (16.9)		
rs4576 (*CTSF*)	AA	238 (43.3)	290 (52.7)	10.35	0.006
	AG	275 (50.0)	224 (40.7)		
	GG	37 (6.7)	36 (6.5)		
rs6913634 (*NT5E*)	CC	467 (84.9)	500 (90.9)	9.31	0.009
	CT	78 (14.2)	47 (8.5)		
	TT	5 (0.9)	3 (0.5)		
rs157906 (*miR-29b*)	GG	365 (66.4)	394 (71.6)	3.62	0.16
	GA	165 (30.0)	138 (25.1)		
	AA	20 (3.6)	18 (3.3)		
rs7781163 (*miR-29b*)	TT	392 (71.3)	400 (72.7)	0.31	0.86
	TC	147 (26.7)	139 (25.3)		
	CC	11 (2.0)	11 (2.0)		
rs2222722 (*miR-30a*)	GG	150 (27.3)	150 (27.3)	0.31	0.86
	GA	286 (52.0)	293 (53.3)		
	AA	114 (20.7)	107 (19.5)		
rs2829801 (*miR-155-5p*)	TT	361 (65.6)	338 (61.5)	2.20	0.33
	TG	172 (31.3)	195 (35.5)		
	GG	17 (3.1)	17 (3.1)		

Table S6. Allele distributions of the seven analyzed SNPs in cases and controls.

SNP (gene)	Allele	Cases, *n* (%)	Controls, *n* (%)	χ²	*P*
rs1048975 (*COPS2*)	G	601 (54.6)	626 (56.9)	1.15	0.28
	T	499 (45.4)	474 (43.1)		
rs4576 (*CTSF*)	A	751 (68.3)	804 (73.1)	6.16	0.013
	G	349 (31.7)	296 (26.9)		
rs6913634 (*NT5E*)	C	1012 (92.0)	1047 (95.2)	9.28	0.002
	T	88 (8.0)	53 (4.8)		
rs157906 (*miR-29b*)	G	895 (81.4)	926 (84.2)	3.06	0.08

	A	205 (18.6)	174 (15.8)		
rs7781163 (*miR-29b*)	T	931 (84.6)	939 (85.4)	0.23	0.63
	C	169 (15.4)	161 (14.6)		
rs2222722 (*miR-30a*)	G	586 (53.3)	593 (53.9)	0.09	0.77
	A	514 (46.7)	507 (46.1)		
rs2829801 (*miR-155-5p*)	T	894 (81.3)	871 (79.2)	1.52	0.22
	G	206 (18.7)	229 (20.8)		

Table S7. Complete conditional logistic regression estimates for all seven SNPs under co-dominant, dominant, and recessive models (adjusted for marital status, education, occupation, and income).

SNP (gene)	Comparison (model)	GC ca/co	Overall GC, aOR (95% CI)	Cardia ca/co	Cardia GC, aOR (95% CI)	Non-cardia ca/co	Non-cardia GC, aOR (95% CI)
rs1048975 (*COPS2*)	GT vs. GG	301/288	1.21 (0.91–1.60)	131/124	1.41 (0.90–2.19)	170/164	1.12 (0.77–1.63)
	TT vs. GG	99/93	1.19 (0.82–1.75)	38/38	1.19 (0.66–2.15)	61/55	1.28 (0.72–1.98)
	GT+TT vs. GG (dominant)	400/381	1.21 (0.92–1.58)	169/162	1.35 (0.88–2.06)	231/219	1.14 (0.79–1.63)
	TT vs. GG+GT (recessive)	99/93	1.05 (0.75–1.47)	38/38	0.96 (0.57–1.61)	61/55	1.11 (0.71–1.73)
rs4576 (*CTSF*)	AG vs. AA	275/224	1.51 (1.16–1.98)	118/98	1.44 (0.97–2.13)	157/126	1.57 (1.08–2.29)
	GG vs. AA	37/36	1.30 (0.78–2.17)	15/15	1.05 (0.46–2.42)	22/21	0.94 (0.46–1.92)
	AG+GG vs. AA (dominant)	312/260	1.48 (1.14–1.92)	133/113	1.39 (0.95–2.03)	179/147	1.56 (1.09–2.24)
	GG vs. AA+AG	37/36	1.05 (0.64–1.73)	15/15	0.89 (0.40–2.00)	22/21	1.19 (0.62–2.58)

	(recessive)						
rs6913634 (*NT5E*)	CT vs. CC	78/47	1.63 (1.08–2.44)	41/20	2.38 (1.27–4.45)	37/27	1.28 (0.74–2.21)
	TT vs. CC	5/3	1.45 (0.31–6.95)	2/0	—	3/3	0.81 (0.14–4.89)
	CT+TT vs. CC (dominant)	83/50	1.62 (1.09–2.40)	43/20	2.51 (1.35–4.67)	40/30	1.24 (0.72–2.11)
	TT vs. CC+CT (recessive)	5/3	1.34 (0.28–6.38)	2/0	—	3/3	0.77 (0.13–4.60)
rs157906 (*miR-29b*)	GA vs. GG	165/138	1.21 (0.91–1.62)	72/55	1.39 (0.88–2.18)	93/83	1.09 (0.74–1.60)
	AA vs. GG	20/18	1.23 (0.79–3.32)	8/5	2.32 (0.64–8.47)	12/13	1.32 (0.54–3.23)
	GA+AA vs. GG (dominant)	185/156	1.25 (0.95–1.65)	80/60	1.43 (0.92–2.23)	105/96	1.12 (0.78–1.60)
	AA vs. GG+GA (recessive)	20/18	1.54 (0.76–3.12)	8/5	1.96 (0.56–6.88)	12/13	1.30 (0.53–3.17)
rs7781163 (*miR-29b*)	TC vs. TT	147/139	0.96 (0.72–1.28)	58/58	0.93 (0.59–1.46)	89/81	1.03 (0.71–1.49)
	CC vs. TT	11/11	0.83 (0.33–2.08)	3/3	0.68 (0.13–3.63)	8/8	0.83 (0.28–2.44)
	TC+CC vs. TT (dominant)	158/150	0.96 (0.72–1.27)	61/61	0.91 (0.58–1.43)	97/89	1.01 (0.70–1.46)
	CC vs. TT+TC (recessive)	11/11	0.84 (0.34–2.09)	3/3	0.70 (0.13–3.70)	8/8	0.82 (0.28–2.38)
rs2222722 (*miR-30a*)	GA vs. GG	286/293	1.03 (0.77–1.38)	115/122	0.97 (0.61–1.56)	171/171	1.07 (0.72–1.58)
	AA vs. GG	114/107	1.29 (0.89–1.87)	56/43	1.80 (1.00–3.25)	58/64	1.00 (0.61–1.65)
	GA+AA vs. GG (dominant)	400/400	1.09 (0.83–1.45)	171/165	1.16 (0.74–1.80)	229/235	1.05 (0.72–1.53)
	AA vs. GG+GA (recessive)	114/107	1.27 (0.92–1.73)	56/43	1.84 (1.11–3.04)	58/64	0.96 (0.63–1.47)
rs2829801 (*miR-155-5p*)	TG vs. TT	172/195	0.84 (0.65–1.10)	71/79	0.79 (0.51–1.21)	101/116	0.87 (0.62–1.22)
	GG vs. TT	17/17	0.85 (0.38–	7/7	0.90 (0.26–	10/10	0.75 (0.26–

		1.87)		3.08)		2.16)
TG+GG vs. TT (dominant)	189/212	0.84 (0.65–1.09)	78/86	0.80 (0.53–1.20)	111/126	0.86 (0.62–1.20)
GG vs. TT+TG (recessive)	17/17	0.89 (0.40–1.95)	7/7	0.94 (0.27–3.20)	10/10	0.79 (0.28–2.25)

ca/co, cases/controls. Wild-type genotypes are the reference (aOR = 1). For rs2829801, genotype labels are harmonized to the TT/TG/GG nomenclature used in Tables S4–S6.

Table S8. Complete joint-effect and multiplicative-interaction estimates for rs4576 (dominant and recessive models) with hard-food preference and pickled vegetable intake in overall and cardia gastric cancer.

Outcome	Dietary factor / model	rs4576 genotype	Exposure	Cases (n)	Controls (n)	aOR (95% CI)
Overall GC	Hard food — dominant	AA	No	60	165	1.00 (Ref.)
		AG+GG	No	73	151	1.16 (0.73–1.85)
		AA	Yes	178	125	3.94 (2.58–6.02)
		AG+GG	Yes	239	109	5.75 (3.78–8.76)
	Interaction: dominant × hard food					1.26 (0.70–2.25)
	Pickled vegetables — dominant	AA	No	79	172	1.00 (Ref.)
		AG+GG	No	117	146	1.71 (1.13–2.59)
		AA	Yes	159	118	3.35 (2.19–5.12)
		AG+GG	Yes	195	114	4.44 (2.92–

						6.74)
	Interaction: dominant × pickled vegetables					0.77 (0.44–1.36)
	Hard food — recessive	AA+AG	No	120	296	1.00 (Ref.)
		GG	No	13	20	1.62 (0.71–3.70)
		AA+AG	Yes	393	218	4.75 (3.42–6.58)
		GG	Yes	24	16	4.42 (2.11–9.26)
	Interaction: recessive × hard food					0.57 (0.19–1.71)
	Pickled vegetables — recessive	AA+AG	No	186	297	1.00 (Ref.)
		GG	No	10	21	0.68 (0.30–1.55)
		AA+AG	Yes	327	217	2.82 (2.09–3.80)
		GG	Yes	27	15	3.28 (1.62–6.67)
	Interaction: recessive × pickled vegetables					NA
Cardia GC[b]	Hard food — dominant	AA	No	29	77	1.00 (Ref.)
		AG+GG	No	35	72	1.16 (0.60–2.22)
		AA	Yes	74	46	4.67 (2.40–9.09)
		AG+GG	Yes	98	41	7.48 (3.83–14.63)
	Interaction: dominant × hard food					1.39 (0.56–3.43)
	Pickled vegetables — dominant	AA	No	35	74	1.00 (Ref.)
		AG+GG	No	52	61	1.79 (0.98–

					3.23)
	AA	Yes	68	49	3.47 (1.83–6.58)
	AG+GG	Yes	81	52	3.60 (1.98–6.53)
Interaction: dominant × pickled vegetables					0.58 (0.25–1.35)
Hard food — recessive	AA+AG	No	61	141	1.00 (Ref.)
	GG	No	3	8	0.77 (0.14–4.24)
	AA+AG	Yes	160	80	5.55 (3.29–9.34)
	GG	Yes	12	7	4.48 (1.48–13.56)
Interaction: recessive × hard food					1.05 (0.14–7.88)
Pickled vegetables — recessive	AA+AG	No	83	129	1.00 (Ref.)
	GG	No	4	6	0.73 (0.18–3.00)
	AA+AG	Yes	138	92	2.75 (1.74–4.33)
	GG	Yes	11	9	1.67 (0.62–4.50)
Interaction: recessive × pickled vegetables					0.84 (0.15–4.73)

[a] All aORs reported in this table were adjusted for marital status, education, occupation, and income.

[b] Several cardia GC strata are based on very few participants (e.g., GG with no hard-food preference: 3 cases and 8 controls); the corresponding estimates are unstable and should be interpreted with caution.
